# Prolonged pemetrexed pretreatment augments persistence of cisplatin-induced DNA damage and eliminates resistant lung cancer stem-like cells associated with EMT

**DOI:** 10.1186/s12885-016-2117-4

**Published:** 2016-02-19

**Authors:** Colin Charles Tièche, Ren-Wang Peng, Patrick Dorn, Laurène Froment, Ralph Alexander Schmid, Thomas Michael Marti

**Affiliations:** Division of General Thoracic Surgery, Inselspital, Bern University Hospital, Department of Clinical Research, University of Bern, Murtenstrasse 50, 3008 Bern, Switzerland

**Keywords:** Pemetrexed, Cisplatin, Chemotherapy, Resistance, Non-small cell lung cancer, Stem cells, EMT

## Abstract

**Background:**

Lung cancer is the leading cause of cancer-related mortality, and new therapeutic options are urgently needed. Non-small cell lung cancer (NSCLC) accounts for approximately 85 % of all lung cancers, with the current standard regimen of care for NSCLC including chemotherapy with pemetrexed as a single agent or in combination with platinum-based agents, e.g. cisplatin. Pemetrexed is a folic acid antagonist that inhibits the synthesis of precursor nucleotides, whereas cisplatin directly induces DNA adducts, the repair of which is dependent on sufficiently high nucleotide levels. In the clinical setting, the pemetrexed-cisplatin combination therapy is administered concomitantly. We hypothesized that prolonged pretreatment with pemetrexed could be beneficial, as prior depletion of nucleotide pools could sensitize cancer cells to subsequent treatment with cisplatin.

**Methods:**

NSCLC A549 and H460 cells were treated with pemetrexed for 72 h. In addition, 24 h of cisplatin treatment was initiated at day 1, 2 or 3 resulting in either simultaneous pemetrexed application or pemetrexed pretreatment for 24 or 48 h, respectively. Cell growth and colony formation as well as senescence induction were quantified after treatment. Cell cycle distribution and phosphorylation of histone variant H2AX as a surrogate marker for DNA damage was quantified by flow cytometry. Relative changes in gene expression were determined by quantitative real time PCR.

**Results:**

Prolonged pemetrexed pretreatment for 48 h prior to cisplatin treatment maximally delayed long-term cell growth and significantly reduced the number of recovering clones. Moreover, apoptosis and senescence were augmented and recovery from treatment-induced DNA damage was delayed. Interestingly, a cell population was identified that displayed an epithelial-to-mesenchymal transition (EMT) and which had a stem cell phenotype. This population was highly resistant to concomitant pemetrexed-cisplatin treatment but was sensitized by pemetrexed pretreatment.

**Conclusions:**

Adaptation of the standard treatment schedule to include pretreatment with pemetrexed optimizes the anticancer efficiency of pemetrexed-cisplatin combination therapy, which correlates with a persistence of treatment-induced DNA damage. Therefore, this study warrants further investigations to elucidate whether such an adaptation could enhance the effectiveness of the standard clinical treatment regimen. In addition, a subpopulation of therapy resistant cells with EMT and cancer stem cell features was identified that was resistant to the standard treatment regimen but sensitive to pemetrexed pretreatment combined with cisplatin.

**Electronic supplementary material:**

The online version of this article (doi:10.1186/s12885-016-2117-4) contains supplementary material, which is available to authorized users.

## Background

Lung cancer is the leading cause of cancer-related mortality, resulting in over 1 million deaths each year worldwide. This is mainly due to the difficulty of early detection and a lack of successful treatment methods, thus more effective therapy options are needed. Non-small cell lung cancer (NSCLC) accounts for approximately 85 % of all lung cancers, and standard chemotherapy for NSCLC includes pemetrexed (multitargeted antifolate, MTA; commercial name ‘Alimta’), as a single agent or in combination therapy (reviewed in [1]). The combination of MTA with cisplatin was recently recommended as the gold standard therapy for adenocarcinoma lung cancer patients with good performance status [2]. In addition, MTA-cisplatin combination therapy is also the recommended treatment regimen for malignant pleural mesothelioma (MPM) (reviewed in [3].

MTA is a folic acid antagonist inhibiting the synthesis of precursor purine and pyrimidine nucleotides required for DNA and RNA synthesis. MTA thereby interferes with the proliferation and survival of replicating cancer cells. Prolonged treatment with MTA induces replicative stress in the form of single stranded DNA, which, if not repaired, can lead to the formation of double stranded DNA breaks [4]. Cisplatin mainly induces intrastrand crosslinks and to a lesser extent also interstrand crosslinks. Repair of intrastrand crosslinks is mainly dependent on functional nucleotide excision repair, which is impaired upon nucleotide depletion [5].

Induction of DNA lesions or DNA replication stress leads to the activation of the DNA damage response (DDR) (reviewed in [6]). During DDR, phosphorylation of histone variant H2AX (γH2AX) serves as a key mediator for the assembly of DNA repair proteins at the sites of DNA damage as well as for the activation of checkpoint proteins. Consequently, analysis of γH2AX is frequently used as a surrogate marker for DDR activation. Prolonged cell cycle arrest after DNA damage induction results at the molecular level in DNA double strand break formation [7] and at the cellular level in a terminal proliferation halt, i.e. senescence [8]. By permanently arresting proliferation of damaged cells, senescence serves as a barrier to cancer development.

An increasing body of evidence suggests that epithelial-to-mesenchymal transition (EMT) plays a crucial role in the tumorigenesis, drug resistance, relapse and metastasis of various cancers (reviewed in [9]). Diverse stimuli can lead to the activation of the EMT signaling cascade thereby inducing the expression of mesenchymal markers, e.g. vimentin. Furthermore, the expression of N-Cadherin is associated with acquisition of EMT phenotype whereas E-cadherin is associated with an epithelial phenotype (reviewed in [9]). Cells can advance only partially through the EMT program, resulting in a mixture of epithelial and mesenchymal traits (reviewed in [9]). For example in breast cancer, a hybrid epithelial/mesenchymal (hybrid E/M) state was identified at the single cell level, which was associated with increased mammosphere formation and poor predicted outcomes in all breast cancer subtypes [10]. In the context of lung cancer, it was shown that IL-6-induced EMT increases adenocarcinoma tumor growth and metastasis *in vivo* [11] and we have recently shown that blocking EMT abrogates resistance to MTA in NSCLC [12]. Mesenchymal cells are characterized by a loss of cell-to-cell contact and a spindle-shaped morphology (reviewed in [13]).

Expression of NANOG, Sox2, CD44 is associated with stemness in various tissues and has allowed the identification of normal stem cells and subsequently also of cancer stem cells (CSCs; reviewed in [9]. For lung cancer, CSCs were identified by means of numerous markers, e.g. drug-resistant side-population, CD133+, ALDH^high^ and EpCAM+ cells (for references, see [14]). However, similar to the latest discoveries concerning the EMT status, more recent findings indicate that increased plasticity might also be present within cancer populations, enabling bidirectional interconvertibility between CSCs and non-CSCs (reviewed in [15]).

In this study, we aimed to optimize the MTA-cisplatin anticancer modality and subsequently performed an in-depth molecular and cellular analysis to elucidate the molecular mechanisms underlying the observed benefit of sequential combination therapy. We demonstrated that prolonged MTA pretreatment improved the combination therapy’s efficiency. This effect correlated with the induction of persistent DNA damage, increased apoptosis and senescence initiation. The occurrence of resistant clones was thereby diminished, however those that did remain featured an epithelial-to-mesenchymal phenotype and were enriched for stem cell traits.

## Methods

### Cell culture and reagents

The NSCLC cell lines A549 (CCL-185) and H460 (HTB-177) were purchased from American Type Culture Collection (ATCC, Manassas, VA, USA) and cultured in Dulbecco’s’ modified Eagle’s medium nutrient mixture F-12 Ham (Cat. #D6421, Sigma-Aldrich, St. Louis, MO, USA), supplemented with 10 % fetal bovine serum (Cat. #10270-106; Life Technologies, Grand Island, NY, USA), 1 % Penicillin/Streptomycin solution (Cat. #P0781, Sigma-Aldrich) and 1 % L-Glutamine (Cat. #25030-024, Sigma-Aldrich) at 37 °C in a humidified 5 % CO_2_ incubator. Cell lines were previously DNA fingerprinted (Microsynth, Bern, Switzerland). Medium was changed every 3 days.

Pemetrexed/MTA (commercial name ‘ALIMTA’; Cat #VL7640) was purchased from Eli Lilly (Suisse) S.A. (Vernier/Geneva, Switzerland). Cisplatin (commercial name ´Cisplatin Ebewe´) was purchased from Sandoz Pharmaceuticals AG (Steinhausen/Cham, Switzerland).

### Drug response and senescence associated β-galactosidase assay

To determine cell growth during the treatment and the initial recovery phase, 1×10^6^ cells were seeded into 150 mm × 20 mm tissue culture treated plates (Cat. #20151, SPL Life Sciences Co., Ltd, Korea). Parallel experiments were performed in triplicate and samples were subsequently processed for flow cytometry as described below. Starting at the day after seeding, i.e. day 0, cells from one plate per treatment were harvested using TrypLE (Cat. #12604021, Invitrogen, Grand Island, NY, USA). Cell titers were determined using a hemocytometer and trypan blue (Sigma-Aldrich) (final concentration 0.1 %) for dead cell exclusion. The cells were washed in phosphate-buffered saline and processed for analysis by flow cytometry as described below. Resistant clones on recovery day 10 were counted on a centered surface of 25 cm^2^, using a 5 mm × 5 mm grid for orientation. To determine cell growth during the extended recovery period, cells were harvested at day 10 of the recovery period, reseeded at a density of 10’000 cells per 150 mm × 20 mm plate and cell titers were subsequently determined as described above. Experiments were repeated independently three times.

Senescent cells were visualized by using the senescence associated β-galactosidase assay (Cat. #20151, Cell Signaling Technology, MA, USA). In detail, 2000 cells were recovered at day 10 after each treatment and were seeded in tissue culture treated 6-well plates, fixed at day 17 and stained overnight according to the manufactures protocol. An inverted light microscope (Eclipse TS100, Nikon Instruments Inc., Melville NA, USA) equipped with a 10x objective was used for visual quantification of senescent cells. Experiments were repeated independently three times.

### Gene expression analysis

Total RNA was isolated and purified with RNeasy Mini Kit (Cat. #74106, Qiagen, Hilden, Germany) and subsequent reverse transcription performed using High Capacity cDNA Reverse Transciption Kit (Cat. #4368814, Applied Biosystems, Foster City, CA, USA) according to the manufacturer’s instructions. Conditions for reverse transcription: 10 min at 25 °C, 120 min at 37 °C, 5 s at 85 °C, hold at 4 °C. Quantitative real-time PCR (qRT-PCR) analyses were performed on a 7500 Fast Real-Time PCR System (Applied Biosystems) with commercially available TaqMan “Assay on Demand” primer/probes (Applied Biosystems) and TaqMan FastUniversal PCR Master Mix (Life Technologies). TaqMan primer list: E-Cadherin (Hs01023894_m1), N-Cadherin (Hs00983056_m1), Vimentin (Hs00185584_m1), CD44 (Hs 01075861_m1 CD44), Oct4B (Hs 00742896_s1 POU5F1), NANOG (Hs04260366_g1), CD133 (Hs 01009254_m1 PROM1), H3F3A (Hs02598544_g1), PP1A (Hs04194521_s1). qRT-PCR cycle conditions were as follows: 50 °C for 2 min, 95 °C for 10 min, 40 cycles of 95 °C for 15 s, 60 °C for 1 min. Gene expression was assessed by ΔΔCt values of the target genes versus control genes H3F3A and PP1A. Experiments were performed as three technical replicates and repeated independently three times.

### Flow cytometry

For analysis by flow cytometry, cells were harvested as described above. Subsequently, cells were washed with phosphate-buffered saline, pH 7.4, fixed and permeabilized with Cytofix/Cytoperm solution (Cat. #554714, BD Biosciences (San Jose, CA, USA)). Staining with mouse Alexa Fluor 488 anti-γH2AX (Ser139) (Cat. #613406, BioLegend, San Diego, CA, USA) antibody was performed in phosphate-buffered saline (Pharmacy, University Hospital Bern, BE, Switzerland) supplemented with 0.5 % saponin (Sigma-Aldrich) and 1 % bovine serum albumin (Sigma-Aldrich) on a rotating wheel (3 rpm) overnight at 4 °C. Subsequently, cells were treated with 100 μg/ml RNase A (Sigma-Aldrich) and DNA was stained simultaneously with 0.5 μg/ml 4’,6-diamidino-2-phenylindole (DAPI) (Sigma-Aldrich). Cell fluorescence was measured on a LSR2 upgraded flow cytometer (BD Biosciences) and analyzed using FlowJo V10 (Tree Star, Inc. (Ashland, OR, USA)). Flow cytometric analysis of the different time points was performed in batches, e.g. cells of the treatment period were analyzed first, followed by the early recovery phase (rec d1-3), recovery day 6 and 10 and at last cells of recovery day 14. Each analysis was accompanied by an untreated control. Approximately 5 % of the cells of the untreated controls were placed in the F/S-high compartment and used as normalization standard to compensate for variations in photomultiplier settings. Buffer treated controls were used to set the gating threshold for γH2AX to 5 % as describe before [16]. When indicated, cells were labeled with 10 mM 5-ethynyl-20-deoxyuridine for 60 min and stained (Alexa Fluor 647) according to the manufacturer’s instructions (C35002; Life Technologies).

To compensate for slight shifts in linear DAPI fluorescence intensity due to treatment-induced changes in FSC/SSC signal intensity, gates for cell cycle distribution analysis were adjusted according to the peak of the G1 subpopulation.

### Statistical analysis

Data are presented as the mean ± standard deviation of at least three independent experiments if not stated differently. Statistical differences were assessed using unpaired *t* test with Welch’s and P values < 0.05 were considered significant.

## Results

### Optimization of treatment schedule potentiates MTA-cisplatin anticancer efficacy

**Fig. 1:**
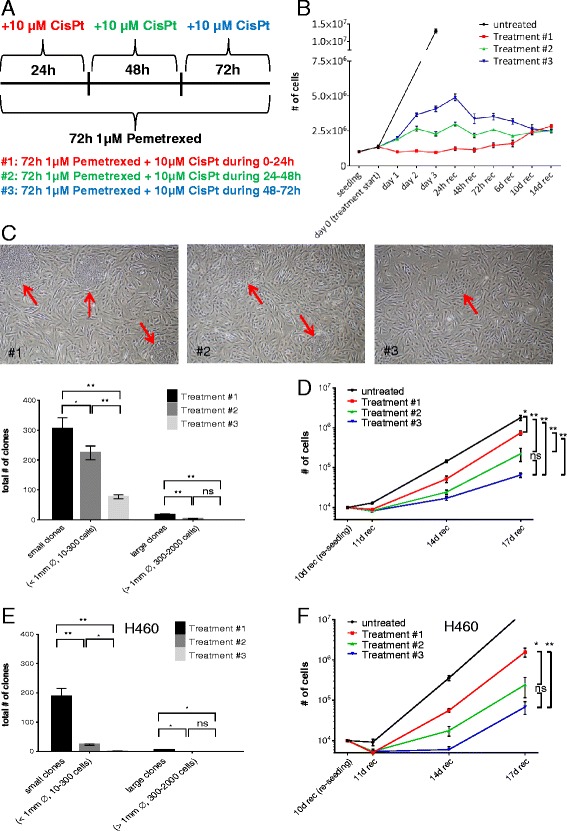
Optimization of the treatment schedule potentiates MTA-cisplatin anticancer efficacy. **a** Schedule of the three tested treatment regimens differing in the duration of MTA pretreatment preceding cisplatin addition (see text for details). **b** Growth curves of A549 cells during the treatment (0-3 days) and early recovery phase (up to 14 days post-treatment). **c** Representative images of large A549 clones (clones are indicated by arrows: treatment #1, 2 clones on the top half of the image; treatment #2, clone on the top half of the image) and small clones (treatment #1 clone on the bottom-right of the image; treatment #2, 1 clone on the bottom half of the image; treatment #3 one small clone visible) at day 10 of the recovery phase. Clones consisting of cells with a small surface area are clearly distinguishable from the background consisting of cells with an enlarged surface area. Quantification of clones was performed as described in the material and methods section. **d** A549 cells exposed to the indicated treatment regimen were harvested at day 10 of the recovery phase, reseeded and cell numbers were determined at the indicated time points. **e** Quantification of colony formation ability of H460 cells as described in *C*. **f** Cell growth of H460 cells during the extended recovery phase as described in *D*. Data represent means of three independent experiments and bars indicate standard deviations. **, *P* < 0.01 and *, *P* < 0.05

Three different treatment regimens were compared to determine whether the effectiveness of the MTA-cisplatin combination therapy is dependent on the treatment schedule. The three regimens consisted of continuous MTA (1 μM) treatment for 72 h (Fig. 1a) in combination with cisplatin (10 μM) treatment for 24 h at different intervals. In detail, cisplatin was applied at 0 → 24 h (treatment #1), 24 → 48 h (treatment #2) or 48 → 72 h (treatment #3) relative to the 72 h of MTA treatment (Fig. 1a). The doubling time (day 0 → 3) of untreated A549 cells was approximately 22 h (Fig. 1b), which is in agreement with the information provided by the American Type Culture Collection. Single MTA treatment (treatment #2, 24 h time point and treatment #3, 24 h and 48 h time points, respectively) decreased cell proliferation compared to untreated cells (Fig. 1b). Cell numbers decreased during the 24 h of concomitant MTA-cisplatin treatment (treatment #1, day 0 → 1) whereas cell numbers initially still increased during MTA-cisplatin co-treatment when preceded by MTA treatment (treatment #2, day 1 → 2 and treatment #3, day 2 → 3). After termination of the chemotherapy regimens (day 3), cell numbers steadily increased from low levels after treatment #1 during the early recovery phase (recovery day 0 → 14) whereas cell growth did not recover after treatment #2 and, starting from recovery day 1, decreased after treatment #3 (recovery day 1 → 14) (Fig. 1b).

At day 10 of the recovery phase, the absolute cell counts were similar among the three different treatment regimens (Fig. 1b). Visual examination of the recovered cells revealed that clones formed by small, cuboid cells could be distinguished from the surrounding monolayer of large cells with a senescent phenotype (discussed below; Fig. 1c). The clones consisting of small cells could be further divided into two categories, i.e. small clones (10–300 cells) and large clones (300–2000 cells). The number of clones differed significantly among the different treatment regimens. In detail, approximately 4 times as many small clones were present after treatment #1 (305 +/-21) compared to treatment #3 (77 +/-3.7; treatment #2 224 +/-13) (Fig. 1c). Of note, numerous large clones were present after treatment #1 (17 +/-1.5) whereas only low numbers of this type were present after treatment #2 (3.3 +/-0.9) and none after treatment #3. Thus, although absolute A549 cell numbers were similar at day 10 of the recovery phase, only treatment #3 efficiently reduced the occurrence of both, small and large recovering clones. Indeed, the increased efficiency of MTA pretreatment on cisplatin toxicity was also confirmed in the NSCLC cell line H460, which also contains an activating KRAS mutation. In detail, at day 10 during the recovery phase, the number of small recovering clones was significantly reduced after treatment #2 (23 +/-1.5) compared to treatment #1 (188 +/-15) and further significantly decreased after treatment #3 (1.0 +/-0.6) (Fig. 1e and Additional file 1: Figure S1). Formation of large H460 clones was reduced after treatment #1 (4.3 +/-0.3) and completely abolished after treatment #2 and #3, respectively. In summary, only treatment #3 efficiently reduced the occurrence of both, small and large recovering clones in both tested NSCLC cell lines.

To evaluate the growth capacity of the remaining cells, the residual cells were harvested at day 10 of the recovery phase and reseeded at low density. All tested treatment regimens reduced plating efficiency compared to the untreated control (day 11) (Fig. 1d). At day 17 of the recovery phase, cell numbers compared to untreated control were 2.4, 8, 27 times lower after treatment #1/2/3, respectively. In other words, long-term cell growth of A549 cells was reduced by a factor of 11 after treatment #3 when compared to treatment #1. Similarly, long-term cell growth of H460 cells was reduced by a factor of 18 after treatment #3 when compared to treatment #1 (Fig. 1f). The doubling time of A549 cells during day 14 → 17 of the recovery phase was comparable after treatment #1, treatment #2 and the untreated control (19, 22.5 and 20 h, respectively), whereas treatment #3 significantly prolonged the doubling time of long-term recovering cells (37 h).

### Prolonged MTA pretreatment and subsequent cisplatin treatment induces senescence

**Fig. 2:**
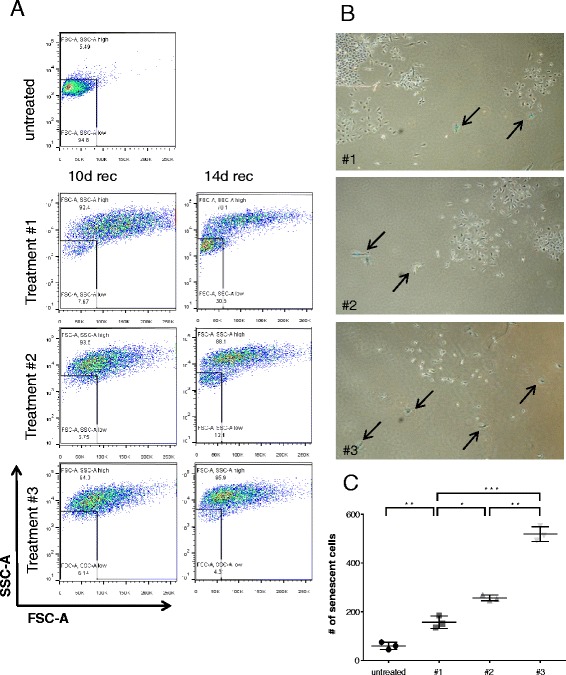
Prolonged MTA pretreatment augments cisplatin-induced senescence in A549 cells. **a** Forward and side scatter analysis by flow cytometry (without reseeding) at day 10 (10d rec) and day 14 (14d rec) of the recovery phase. **b** Representative images of cells acquired by phase contrast-based microscopy at day 17 of the recovery phase (reseeded at day 10). Arrows indicate cells which stain positive for senescence associated β-galactosidase activity. **c** Quantification of senescent cells based on increased β-galactosidase activity **b**. Represented are three independent experiments and bars indicate means and standard deviations. ***, *P* < 0.001, **, *P* < 0.01 and *, *P* < 0.05

As mentioned above, visual examination revealed that the cells surrounding the recovering clones displayed morphologic changes that are associated with senescence, namely increased cell size and flattened shape (Fig. 1c; reviewed in [17]). Detectable by flow cytometry, increased forward (cell size) and side (cellular granularity) scatter intensity (F/S-high; Fig. 2a and Additional file 2: Figure S2) is an additional characteristic associated with senescence (reviewed in [18]). Flow cytometric analysis at day 10 and 14 of the recovery phase (without reseeding) revealed that the highest ratio of F/S-high versus F/S-low cells was observed after treatment #3. In detail, the ratios of FS-high versus FS-low cells were 12, 14 and 15.5 after day 10 and 2.3, 7.3 and 24 after day 14 for treatment #1/2/3, respectively. In other words, at day 14 of the recovery phase (without reseeding), a significant fraction of cells after treatment #1 restored normal forward and side scatter intensity (F/S-low), which was less pronounced after treatment #3 (31 % versus 7 %, respectively, *p* > 0.01). To quantify senescence induction, cells from recovery day 10 were reseeded at low density. At day 17 of the recovery phase, quantification of senescence-associated β-galactosidase (SA-β-Gal) activity revealed that the fraction of SA-β-Gal-positive cells (indicated by black arrows in Fig. 2b) was 3.5-fold higher after treatment #3 compared to treatment #1 (Fig. 2c). However, visual examination revealed that a fraction of the cells was clearly proliferating (see also Fig. 1d) giving rise to distinct colonies as described below.

### Prolonged MTA pretreatment exacerbates cisplatin-induced cell cycle arrest

Terminal cell cycle arrest is a classic hallmark of senescence, and has been observed after treatment with chemotherapy (reviewed in [17]). Therefore, we monitored the cell cycle distribution of A549 cells during and after combined treatment with MTA and cisplatin. It was shown previously that the increase in cell size of A549 cells after cisplatin treatment leads to a shift of the characteristic G1-phase peak in the DNA histogram plot, and an even more pronounced shift for the G2/M-phase peak [19]. In agreement with these findings, already at day 1 after treatment #1 the DAPI signal intensity of F/S-high cells was increased compared to F/S-low cells (Additional file 3: Figure S3). Thus, the populations consisting of F/S-high cells and F/S-low cells were analyzed separately in our subsequent analyses (Additional file 3: Figure S3). In our initial experiments, we determined the cell cycle distribution by analyzing the incorporation of 5-ethynyl-2’-deoxyuridine (EdU), a nucleoside analog of thymidine, which is incorporated into DNA during replication. Treatment of A549 cells with 1 μM MTA for 24 h or 48 h reduced the fraction of replicating cells, i.e. EdU-positive cells, to 4.6 % and 0.7 %, respectively, compared to 42 % in control cells (Additional file 4: Figure S4). This is consistent with an earlier report showing that treatment with 200 nM MTA for 24 h nearly completely abolished incorporation of thymidine analogues in colorectal adenocarcinoma cells [20]. However, it is challenging to accurately set the gates of the cell cycle phases on a DNA histogram blot from a cell population with an irregular cell cycle distribution, e.g. after MTA treatment, without being able to identify replicating cells by EdU incorporation. Our previous findings revealed that H2AX phosphorylation levels after DNA damage induction were enhanced during S-phase compared to the G1-phase levels [21]. Hence, H2AX phosphorylation was plotted over DNA content as a “zebra blot” (a feature of the software FlowJo) (Additional file 5: Figure S5), which allowed us to more accurately determine the G1 to S- and the S to G2/M-borders of the cell cycle phases from cell populations with irregular cell cycle distributions, e.g. after drug treatment (Additional file 6: Figure S6).

**Fig. 3:**
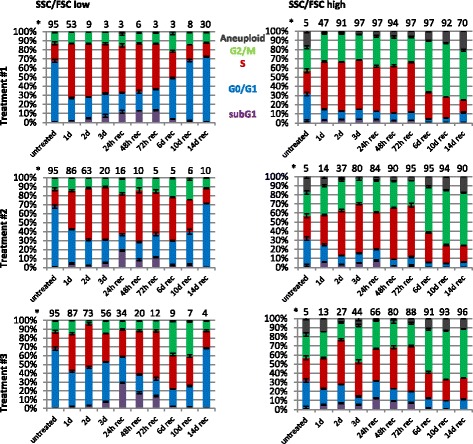
Prolonged MTA pretreatment exacerbates cisplatin-induced cell cycle arrest and reduces the fraction of recovering cells. Flow cytometric analysis was performed at the indicated time points and subpopulations featuring either increased forward and side scatter intensity (F/S-high) or normal forward and side scatter intensity (F/S-low) were identified as indicated in Figure S2. Cell cycle analysis was performed as indicated in Figure S6. Data shown are the mean values and standard deviations of three experiments. *Percentage of total cell population (mean from three experiments)

During the initial 72 h treatment phase, the fraction of F/S-high cells was increased by all tested treatment regimens although with different frequencies (Fig. 3 and Additional file 2: Figure S2). MTA-cisplatin co-treatment for 24 h (treatment #1, day 0 → 1) resulted in a rapid depletion of F/S-low cells and accumulation of the remaining F/S-low cells in S-phase, which was also observed in the corresponding F/S-high cell fraction (47 %; Fig. 3). In contrast, the fraction of F/S-high cells was significantly less increased after MTA-alone treatment for 24 h [treatment #2&3, day 0 → 1 (14 % and 13 %)] or 48 h [treatment #3, day 0 → 2 (27 %)]. MTA-alone treatment for 24 h resulted in an early S-phase arrest of F/S-low cells, whereas an accumulation in S-and G2/M-phase was detectable in F/S-high cells. MTA-alone treatment for 48 h increased the fraction of F/S-low cells in S-phase, which was even more pronounced for F/S-high cells. Thus, at the start of the cisplatin addition during treatment #1 (untreated cells), 95 % of the cells featured a F/S-low phenotype and mainly resided in the G1-phase of the cell cycle. In contrast, at the start of cisplatin addition during treatment #3 (day 2), 27 % of the cells had a F/S-high phenotype and the majority of those cells were arrested in S-phase. Interestingly, common to all tested treatment regimens, a significant increase in the fraction of F/S-high cells was observed at the end of the 24 h cisplatin treatment. Also common to all treatments, cisplatin exposure triggered cell cycle progression, e.g. during treatment #1, cells advanced from G1- to S-phase whereas S-phase cells progressed to G2/M-phase during treatment #3. At the end of treatment #1 (day 3), 97 % of the cells featured a F/S-high phenotype and most cells were arrested in S-phase. However, after treatment #3, only 44 % of the total remaining cells featured a F/S-high phenotype and a prominent arrest in the G2/M-phase was observed in this subpopulation.

During the recovery phase from treatment #1, the fraction of F/S-low cells steadily increased up to day 14 (30 %) and this subpopulation featured a normal cell cycle distribution. However, the majority of cells (70 %) maintained F/S-high phenotype at day 14 of the recovery phase, an effect which was even more pronounced after treatment #2&3 (90 % and 96 %). 14 days after completion of treatment #1, the majority of the F/S-high cells were in the G2/M-phase (55 %) or acquired a DNA content larger than G2/M, i.e. an aneuploid DNA content (20 %). The majority of the cells were also arrested in the G2/M phase (55 %) 14 days after completion of treatment #3. However, the fraction of F/S-high cells with aneuploid DNA content (11 %) was ~2-fold reduced compared to treatment #1 (Fig. 3). In summary, recovery of F/S-low fraction featuring a normal cell cycle distribution was maximally delayed after treatment #3 and the remaining F/S-high cells (day14 rec) were primarily arrested in the G2/M-phase.

Cell cycle analysis also revealed the appearance of a fraction of cells containing sub-G1 DNA content, which is a hallmark of cells undergoing apoptosis (reviewed in [22]. Most sub-G1 cells were observed 24 h after treatment end (rec d1) in the F/S-low fraction, reaching about 10 % for treatment #1, 19 % after treatment #2 and 29 % after treatment #3. The higher frequency of apoptotic cells after treatment #3 is in agreement with the cell growth analysis (Fig. 1b), which indicates a steady decrease in cell numbers during the recovery phase after treatment #3 but not after treatment #1 & #2.

### Prolonged MTA pretreatment results in persistent DNA damage accumulation

**Fig. 4:**
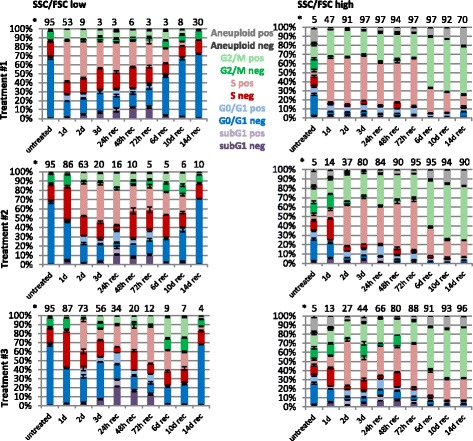
Prolonged MTA pretreatment enhances cisplatin-induced accumulation of persistent DNA damage. Basal H2AX phosphorylation was set at 5 % in untreated controls and used for normalization among experiments as described in the material and methods section. Cell cycle phase-specific H2AX phosphorylation levels were determined as described in Figure S5. Data shown are the mean values and standard deviations of three experiments. *Percentage of total cell population (mean from three experiments). neg = γH2AX negative; pos = γH2AX positive

We have previously demonstrated that accumulation of persistent DNA damage leads to a cell cycle arrest and induction of senescence in A549 cells [23]. Thus, we determined the effect of the different treatment regimens on H2AX phosphorylation, a marker of DNA damage. After MTA-cisplatin co-treatment (treatment #1), H2AX was rapidly phosphorylated (day 1) in the majority of F/S-low cells during the S- and G2/M-phase, to a lesser extend in G1 cells, which was even more pronounced in F/S-high cells (Fig. 4). MTA-alone treatment for 24 h (treatment #2&3, day 1) did not result in robust H2AX phosphorylation in F/S-low cells whereas approximately one third of the F/S-high cells in the S- and half of G2/M-phased stained positive for γH2AX. However, MTA treatment for 48 h (treatment #3, day 2) further increased H2AX phosphorylation in the remaining F/S-low fraction and H2AX phosphorylation was increased in nearly 100 % of the F/S-high cells. Surprisingly, subsequent MTA-cisplatin co-treatment for an additional 24 h (treatment #3, day 3) reduced the fraction of γH2AX positive cells in both the F/S-low and F/S-high subpopulations compared to 48 h MTA-alone treatment (treatment #3, day 2).

During the recovery from treatment #1, H2AX phosphorylation in the F/S-low cells steadily decreased in all cell cycle phases, reaching nearly basal levels by day 10. In contrast, 10 days after treatment #3, H2AX phosphorylation was still detectable in over 50 % of the S/G2/M-phase cells of the F/S-low subpopulation. Strikingly, in all three F/S-high subpopulations, γH2AX phosphorylation was still increased in more than 90 % of the cells at day 14 of the recovery phase (Fig. 4). Therefore, treatment #3 was the most effective at inducing long term DNA damage after an extended recovery phase.

### Therapy-resistant lung cancer cells feature stem-like and EMT properties

**Fig. 5:**
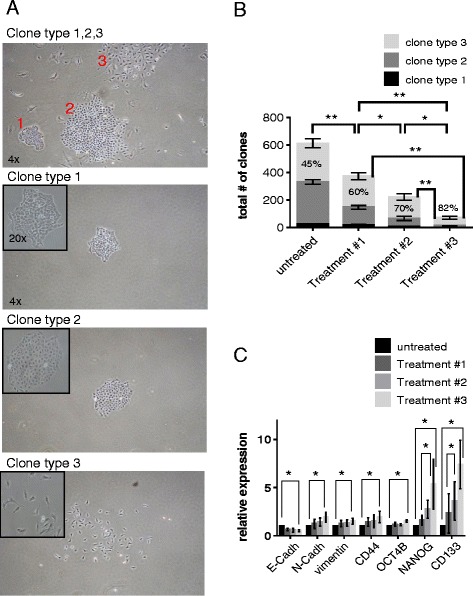
Therapy-resistant lung cancer cells featuring stem-like and EMT properties are most vulnerable to treatment #3. **a** Untreated A549 cells were seeded at low density and colony formation was subsequently analyzed by phase-contrast microscopy after 7 days. Colonies were clustered into three distinct types, see text for details. **b** Quantification of colony formation at day 17 of the recovery phase. Data shown are the mean values and standard deviations of three independent experiments. **, *P* < 0.01 and *, *P* < 0.05. **c** Analysis of gene expression by rtPCR. Data shown are the mean values and standard deviations of three independent experiments. *, *P* < 0.05

As described above, we found that prolonged MTA pre-treatment (treatment #3) significantly augmented the inhibitory effect of cisplatin on lung cancer cell growth compared to co-treatment (treatment #1; Fig. 1a) even during the extended recovery phase (Fig. 1d). At day 17 of the recovery phase, colonies consisting of dividing cells were detectable after all tested treatment regimens, although with different frequencies. In detail, compared to the untreated control, colony formation was reduced by factor of 1.7, 2.8 and 8.9 after treatment #1/#2/#3, respectively (Fig. 5), which is in agreement with the reduction in cell numbers reported above (Fig. 1d). Visual examination revealed that the morphology of the therapy-resistant colonies and the cells therein was heterogeneous. However, we were able to cluster the colonies into three distinct types based on morphological differences (Fig. 5a). Resistant colonies characterized by tight/continuous boarders, comprising cells with an embryonic stem cell-like phenotype, e.g. high nucleus to cytoplasm ratio, were categorized as type 1 colonies. Colonies consisting of cells with a low nucleus to cytoplasm ratio, which were not so tightly packed but still featured continuous boarders, were categorized as type 2 colonies. Colonies without a continuous boarder consisting of elongated cells were categorized as type 3 colonies. Interestingly, visual examination revealed that untreated A549 cells seeded at a low density give rise to colonies with distinct morphological features with a frequency of 5 %, 50 % and 45 % for colony type 1, 2 and 3, respectively (Fig. 5b). After treatment #1, the relative fraction of type 3 clones was increased in the therapy-resistant population (60 %), which was even further augmented after treatment #2&3 (70 % and 82 %, respectively). However, the absolute number of colonies was decreased by all three treatment regimens, with a 5.4-fold reduction after treatment #3 compared to treatment #1.

We have previously shown that EMT plays a crucial role in lung cancer chemotherapy resistance [12]. Therefore, we further determined the expression of EMT marker genes in the therapy-resistant cells at day 17 of the recovery phase (Fig. 5c). Expression of the epithelial marker E-cadherin was decreased in therapy-resistant cells after all treatment regimens whereas expression of genes associated with a mesenchymal phenotype, e.g. N-cadherin and vimentin was increased, and was most pronounced after treatment #3 (Fig. 5c). In agreement, most of the therapy-resistant colonies after treatment #3 featured a mesenchymal phenotype (Fig. 5a and b), i.e. loss of cell-to-cell contact, and they contained spindle-shaped cells, a characteristic feature of mesenchymal cells (reviewed in [13]).

The presence of cancer stem cells is also associated with chemotherapy resistance [24, 25]. We determined mRNA expression levels of the stemness genes *NANOG* and *Oct4B*, and also of the putative lung cancer stem cell markers CD44 and CD133 (Fig. 5c). The expression of all stem cell markers was increased in the therapy-resistant cells after treatment #1, further enhanced after treatment #2 and most augmented after treatment #3. Of note, the expression of NANOG and CD133 was 2.5 and 3 fold increased after treatment #3 compared to treatment #1, respectively.

In summary, we provide evidence that the inhibitory effect of MTA-cisplatin combination therapy on lung cancer cell growth can be further augmented by the optimization of the treatment schedule. Prolonged MTA pretreatment preceding cisplatin treatment reduces cell growth and colony formation compared to concomitant treatment, increases the fraction of senescent cells and decreases the occurrence of aneuploid cells. Furthermore, our investigations reveal that prolonged MTA-pretreatment enhances treatment-induced cell cycle arrest and significantly delays recovery after DNA damage induction. Finally, we have demonstrated that treatment-resistant colonies display a mesenchymal-like morphology (colony type 3), with elevated expression of stem cell and EMT-associated genes. But pretreatment with MTA prior to cisplatin reduces the number of these colonies compared to the standard concomitant treatment.

## Discussion

A recent retrospective analysis of three phase III randomized controlled trials indicated that concomitant MTA-cisplatin combination therapy should be considered as the gold standard treatment for advanced adenocarcinoma (reviewed in [1]). The present *in vitro* study provides evidence that suggests that pretreatment with MTA prior to cisplatin is significantly more efficient than concomitant treatment with both drugs. These results are in agreement with the study of Kano et al., which revealed that exposure of A549 cells to MTA for 24 h and subsequent cisplatin treatment for 24 h followed by a 72 h recovery phase synergistically reduced cell survival whereas the inversed treatment regimen or the simultaneous application of MTA and cisplatin resulted in antagonistic effects [26]. Similarly, 24 h pretreatment with MTA sensitized A549 cells to a subsequent treatment with a histone deacetylase inhibitor and increased the survival-benefit of the combination treatment in a patient-derived lung cancer mouse xenograft model [27]. In addition, MTA pretreatment followed by treatment with a protein kinase Cβ inhibitor was shown to synergistically reduce cell growth of four NSCLC cell lines whereas the inverse treatment schedule had an antagonistic effect [28]. Thus, the present results are in agreement with the general consensus in the literature indicating that MTA pretreatment sensitizes NSCLC cells to a variety of cytotoxic drugs. The present study provides for the first time an in-depth analysis of the effects on NSCLC cells of the combined treatment of MTA and cisplatin over an extended recovery period.

24 h after the end of the treatment phase, cell numbers were ~5-fold lower after treatment #1 compared to treatment #3. Although a short-term recovery analysis would therefore suggest treatment #1 to be the most efficient anticancer treatment, treatment #3 was more effective over a more extended recovery period. Tumor growth in animal models is usually monitored over periods of several weeks. However, further studies will be needed to demonstrate the superior anticancer efficiency of treatment #3 *in vivo*.

Controversial results concerning the effect of cell density, e.g. gap junction intercellular communication, on survival of cancer cells after treatment with DNA damaging agents including cisplatin are reported in the literature [29–31]. At the start of the 24 h cisplatin treatment, cell density was 2.7-fold higher after extensive pemetrexed pretreatment (treatment #3, day 2) compared to treatment #1 (day 0) (Fig. 1b). However, cell density was still >4 times below full confluence (Fig. 1b, untreated control, day 3), which is associated in NSCLC cell lines with DNA damage resistance [29]. Hence, an alternative experimental setting will be necessary to elucidate if survival after pretreatment with MTA prior to cisplatin challenge is significantly altered by variations in cell culture density.

While increased senescence was observed after treatment #1, as demonstrated by increased β-galactosidase activity, cell size and granularity, these effects were much more pronounced after treatment #3. Consistent with these findings of senescence induction, it has been shown that MTA-treated malignant mesothelioma cells also undergo accelerated senescence [32]. Interestingly, the same study revealed that conditioned media from senescent MPM cells triggered the emergence of EMT-like, clonogenic and chemoresistant cell subpopulations. Senescent cancer cells can be cleared by the immune system, however the role of senescence in cancer progression is still debated (reviewed in [17]). Senescence serves as a physiological barrier against tumor initiation and progression. On the other hand, senescent cancer cells might be able to overcome their dormant state representing a dangerous potential for tumor relapse. Thus, it will be crucial to utilize an immune competent animal model to determine the schedule-dependent anticancer efficiency of the combination therapy.

We observed that the three tested treatment regimens predominantly induced senescence rather than apoptosis. In contrast, Yang et al. [33] showed that 60 % of A549 cells became apoptotic after treatment with 1 μM MTA for 72 h. Of note is that, the present study estimated the fraction of apoptotic cells based on the sub-G1 DNA content determined by flow cytometry whereas Yang et al. performed their analysis by comet assay and quantification of caspase activity.

It is well-established that the cell cycle status plays a critical role in the efficiency of combination chemotherapy. Unperturbed cells are maximally sensitive to cisplatin treatment during late G1/early S-phase and least sensitive during peak DNA synthesis (reviewed in [34]). We showed that treatment with 1 μM MTA alone for 24 h resulted in the accumulation of cells in S-phase, which is in agreement with a previous study [33]. At the start of the 24-h cisplatin treatment, the fraction of F/S-low cells in S-phase was highest in treatment #3 (day2) compared to treatment #2 (day1) and treatment #1 (untreated). The effect was even more pronounced in F/S-high cells. In agreement with our observation, Yang et al. also reported a significant S-phase arrest of A549 cells after treatment with 1 μM MTA for 48 h [35]. Thus, we observed increased cisplatin sensitivity upon MTA-induced S-phase arrest, suggesting that it is not the S-phase status *per se* but is more likely to be the MTA-induced perturbation of DNA synthesis, which sensitizes cells to subsequent cisplatin treatment. Indeed, it has been reported that cells blocked at the G1/S-boundary remained sensitive to cisplatin after release of the block [36].

Comprehensive analysis revealed that 10 days after treatment #1, the cell cycle distribution of the F/S-low subpopulation was similar to the untreated control whereas cells were mainly arrested in S/G2/M-phase after treatment #3. Notably, at day 14 of the recovery phase, all F/S-low subpopulations displayed normal cell cycle distribution after the three treatment regimens although this fraction was significantly lower after treatment #3. In summary, during the extended recovery period, a subpopulation of cells in all three treatment groups overcomes cell cycle arrest and successfully completes mitosis, as also indicated by the presence of proliferating clones at later recovery time points. Thus, this small fraction of cells is resistant to even the most efficient treatment regimen tested in this study, even though it is smaller in number than for other treatments.

It has been previously shown that aneuploidy is associated with poor prognosis in NSCLC [37, 38]. Interestingly, 14 days after treatment #1&#2, a significant fraction of (p53-proficient) A549 cells acquired abnormal chromosome content, i.e. became aneuploid, which was less pronounced after treatment #3. We previously observed that induction of persistent DNA damage in p53-deficient HCT116 cells due to REV3 inhibition results in prolonged mitosis and subsequent return to interphase without cell division resulting in aneuploidy [23], which was not observed in p53-proficient HCT166 cells nor A549 cells, independently of p53 status. Thus, results from the present study suggest that concomitant MTA-cisplatin treatment might have an adverse effect on the protective function of p53 upon the induction of aneuploidy. Treatment #3 not only significantly reduced the number of surviving cancer cells but also decreased the fraction of aneuploid cells in the remaining, therapy-resistant population.

Treatment of A549 cells with 1 μM MTA for 24/48 h led to a time dependent increase in H2AX phosphorylation, which is in agreement with the literature [33, 35]. Simultaneous MTA-cisplatin treatment considerably increased H2AX phosphorylation. Interestingly, after MTA pretreatment for 24 h (treatment #2, day1), subsequent cisplatin-MTA treatment for 24 h (treatment #2, day2) did not further increase H2AX phosphorylation levels compared to MTA treatment alone (treatment #3, day2). Furthermore, after MTA pretreatment for 48 h (treatment #3, day2), subsequent cisplatin-MTA treatment for 24 h (treatment #3, day3) resulted in decreased H2AX phosphorylation levels in F/S-low and also F/S-high cells independently of the cell cycle status. In this context, it has been shown that nucleotide depletion leads to sequestration of replication protein A (RPA), thereby restricting its availability for nucleotide excision repair [5], the primary DNA repair system processing cisplatin adducts. Thus, we hypothesize that MTA-induced nucleotide depletion induces the sequestration of RPA to single stranded DNA formed at stalled replication forks. Insufficient RPA levels limit nucleotide excision repair of cisplatin adducts and subsequent H2AX phosphorylation [21]. Additional cisplatin treatment may overload molecular pathways required for the maintenance of the high γH2AX levels induced by MTA pretreatment, but further experiments will be required to elucidate the exact molecular mechanisms involved.

An initial increase in γH2AX phosphorylation was observed during the recovery phase after treatment #3. In this context, it has been shown that reduced nucleotide levels in combination with cisplatin treatment lead to nucleotide excision repair-mediated single-strand gaps, which are likely to be converted into double-strand breaks in the subsequent S-phase [39]. Similarly, treatment with 5-Fluorouracil leads to the incorporation of 5-Fluorouracil and uracil during S-phase generating DNA repair-dependent, persistent DNA strand breaks during the successive G2/M-G1-phase, thereby interfering with the replication machinery in the subsequent S-phase [40]. Thus, the increased levels of H2AX phosphorylation at the extended recovery time points after treatment #3 might be due to the persistence of complex DNA damage or repair intermediates. In this context, it has been shown before that the persistence of H2AX phosphorylation 24 h after cisplatin treatment correlated with the loss of clonogenic potential [41]. In summary, the analysis of H2AX phosphorylation levels have provided a first insight into the molecular mechanisms underlying the increased efficiency of the combination therapy after prolonged MTA pretreatment. Further studies will be necessary to elucidate the exact nature of the resulting DNA damage after prolonged MTA pretreatment. It has been reported that cell lines contain subpopulations, which can be identified based on various features. For example, from parental A549 cells, a subpopulation with increased sphere formation capacity has been identified based on co-expression of EpCAM/CD166/CD44 [42]. The current experiments revealed the existence of three distinct subpopulations in parental A549 cells by virtue of their capacity to give rise to colonies with divergent morphologies, and we focused on resistant cells surviving the combination treatment. Although the absolute numbers of all the clone types decreased after combination therapy, type 3 clones characterized by an enlarged cell size and mesenchymal morphology were relatively enriched, most prominently by treatment #3. Formation of type 1&2 clones was almost completely abolished by treatment #3. Further analysis of the subpopulation surviving treatment #3 revealed not only the expression of EMT markers but also that of stemness genes was increased indicating that the resistant clones have characteristic features associated with hybrid-E/M state. In agreement with these findings, it has been shown that cisplatin-resistant NSCLC sublines displayed a putative stemness signature with increased expression of CD133/CD44 compared to the corresponding parental cells [43]. Interestingly, expression of stem cell markers NANOG, Oct4 and Sox2 were significantly upregulated in those cells as were the EMT markers c-Met and Beta-catenin, which is in agreement with a hybrid E/M state. Further, a hybrid-E/M state was also identified in tumors of different origin, e.g. breast cancer [10] and clear cell renal cell carcinoma [44]. Since the hybrid-E/M state was associated with poor survival in breast cancer [10], it is tempting to speculate that the hybrid-E/M state might allow tumor cells to more readily differentiate either to a more mesenchymal phenotype (associated with invasiveness, metastasis and chemoresistance) or an epithelial phenotype (associated with metastatic colonization/proliferation) [45] depending on the demands of the tumor microenvironment [44]. A recent publication revealed the existence of a hybrid-E/M state in various lung adenocarcinoma cell lines [46]. Lung adenocarcinoma cell lines containing a significant fraction of hybrid-E/M cells were highly invasive despite gene and protein expression of epithelial markers. It was proposed that the hybrid-E/M state is controlled by a regulatory network consisting of mir-200/Zeb and Snail/miR34 [47]. Thus, our study adds to the growing body of literature identifying the hybrid-E/M state and its regulatory switch as a potential therapeutic target for cancer therapy (reviewed in [48]). From a pragmatic point of view, we established in this study a protocol to efficiently select a subpopulation consisting of highly resistant lung cancer cells characterized by a hybrid-E/M state, which, at least in breast cancer, is associated with poor survival. Additional experiments will be necessary to elucidate whether the increase in cells featuring a hybrid-E/M state is due to a treatment-induced selection process or if the treatment regimen induces gene expression changes in clone type 3 cells, e.g. increased expression of stem cell genes.

Our study was limited to the analysis of the cell line A549 containing an activating mutation of the KRAS oncogene. In lung adenocarcinoma, oncogenic KRAS mutations are highly prevalent (~25 %) but therapy choices are very limited (reviewed in [49]) indicating that our findings might be of relevance to improve treatment of a significant fraction of lung adenocarcinoma patients. However, further analysis of cell lines and primary cultures containing alternative mutational signatures will be necessary to evaluate if these findings are also of relevance for different lung cancer subsets.

Currently, MTA is administered as a daily 10-min infusion, which results in a relative rapid clearance from the body. However, MTA is rapidly converted intracellularly to its active form by polyglutamation. Thus, active MTA polyglutamate derivatives are sustained in tumor cells long after the MTA blood levels have declined, explaining the clinical efficiency of the initial phase I trials with single-agent MTA therapy, which was administered on day 1 every 21 days for up to 6 cycles (reviewed in [50]). The present study reveals that prolonged MTA pretreatment increases the efficiency of the combination therapy *in vitro*. Hence, it is tempting to speculate that a delayed cisplatin administration might also increase the efficiency of the MTA-cisplatin combination therapy in the clinical setting.

### Conclusions

The present study has revealed that the efficiency of the MTA-cisplatin combination therapy can be augmented *in vitro* by optimizing the treatment schedule. The increased efficiency after prolonged MTA pretreatment is attributed to the induction of persistent DNA damage, which in turn results in increased apoptosis and senescence initiation thereby ultimately diminishing the occurrence of resistant clones. Therefore, our study warrants further experiments to elucidate whether an optimization of the standard therapy schedule might also potentiate the current combination treatment regimen *in vivo*. In addition, the remaining, therapy resistant cells not only exhibited a mesenchymal phenotype but also featured an increased expression of stem cell markers, both distinct hallmarks of a hybrid-E/M state. Thus, our research is the starting point for future in-depth analysis of this therapy-resistant subpopulation in lung cancer.

## Additional files

Additional file 1: Figure S1. Optimization of the treatment schedule potentiates MTA-cisplatin anticancer efficacy. Representative image of a large H460 clone (indicated by the arrow after treatment #1) and a small clone (indicated by the arrow after treatment #2) at day 10 of the recovery phase. Quantification of clones was performed as described in the material and methods section. (PPTX 230 kb) Additional file 2: Figure S2. Flow cytometric analysis of forward (cell size) and side (cellular granularity) scatter intensity as an alternative readout for senescence. Approximately 5 % of the cells of the untreated controls were placed in the F/S-high compartment and used as normalization standard as described in the material and methods section. Forward and side scatter analysis by flow cytometry (without reseeding) at the indicated time points during the treatment and recovery phase. Shown are representative images of three experiments. (PPTX 389 kb) Additional file 3: Figure S3. MTA-cisplatin treatment leads to increased cell size (FSC) and granularity (SSC) thereby influencing DAPI signal intensity. A549 cells were concomitantly treated with MTA-cisplatin for 24 h and subsequently analyzed by flow cytometry. ***A*** Subpopulations featuring either normal forward and side scatter intensity (F/S-low) or increased forward and side scatter intensity (F/S-high) are indicated by the blue and red gates, respectively. ***B*** Histogram blots of DNA content from F/S-low and F/S-high subpopulations. Specific cell cycle phases of the F/S-low and F/S-high subpopulations are indicated by the blue and red gates, respectively. ***C*** Cell cycle phase-specific H2AX phosphorylation was determined by the same cell cycle gating strategy as shown in *B* in combination with applying the 5 % threshold for basal H2AX phosphorylation levels as indicated in the material and methods section. (PPTX 376 kb) Additional file 4: Figure S4. MTA treatment abolishes DNA replication. Treatment of A549 cells with 1 μM MTA for the indicated time points. DNA replication, indicated by EdU incorporation, and cellular DNA content (DAPI) were detected simultaneously by flow cytometry. (PPTX 62 kb) Additional file 5: Figure S5. Applied strategy to determine cell cycle phases and cell cycle phase-specific H2AX phosphorylation levels by flow cytometry. To determine more accurately the G1 to S- and the S to G2/M-borders of the cell cycle phases from cell populations with irregular cell cycle distributions, H2AX phosphorylation over DNA content was blotted as “zebra blots” as described in the text. Shown are specific cell cycle gates indicated in blue for the F/S-low subpopulations of untreated controls. Specific cell cycle gates indicated in red are shown for the F/S-high subpopulations of treated samples. A 5 % threshold for basal H2AX phosphorylation levels was applied as indicated in the material and methods section. Analysis was performed after 24 h of MTA and cisplatin co-treatment, e.g. treatment #1, day 1. Data shown are representative of three experiments. (PPTX 104 kb) Additional file 6: Figure S6. Applied strategy to determine cell cycle phases of subpopulations by flow cytometry. Flow cytometric analysis was performed at the indicated time points and subpopulations featuring either increased forward and side scatter intensity (F/S-high) or normal forward and side scatter intensity (F/S-low) were identified as indicated in Supplementary Figure S3. Cell cycle analysis was performed independently for F/S-low and F/S-high cells. Gates set to determine the cell cycle distribution of F/S-low and F/S-high subpopulations were determined as described in Figure S5 and are indicated in blue and red, respectively. Indicated are days (d) during treatment and the recovery phase (rec d). Data shown are representative of three experiments. (PPTX 386 kb)

## Abbreviations

CSCs: cancer stem cells
DAPI: 4’,6-diamidino-2-phenylindole
DDR: DNA damage response
EdU: 5-ethynyl-2’-deoxyuridine
EMT: epithelial-to-mesenchymal transition
GLDC: glycine decarboxylase
hybrid E/M: hybrid epithelial/mesenchymal
MPM: malignant pleural mesothelioma
MTA: pemetrexed
NSCLC: non-small cell lung cancer
RPA: replication protein A
TYMS: thymidylate synthase
γH2AX: phosphorylated histone variant H2AX
