# Supplementary figures and images for: Prolonged pemetrexed pretreatment augments persistence of cisplatin-induced DNA damage and eliminates resistant lung cancer stem-like cells associated with EMT
Source: BMC Cancer. 2016 Feb 19;16:125. doi: 10.1186/s12885-016-2117-4 (PMC4759918; doi:10.1186/s12885-016-2117-4)

## Slide 1
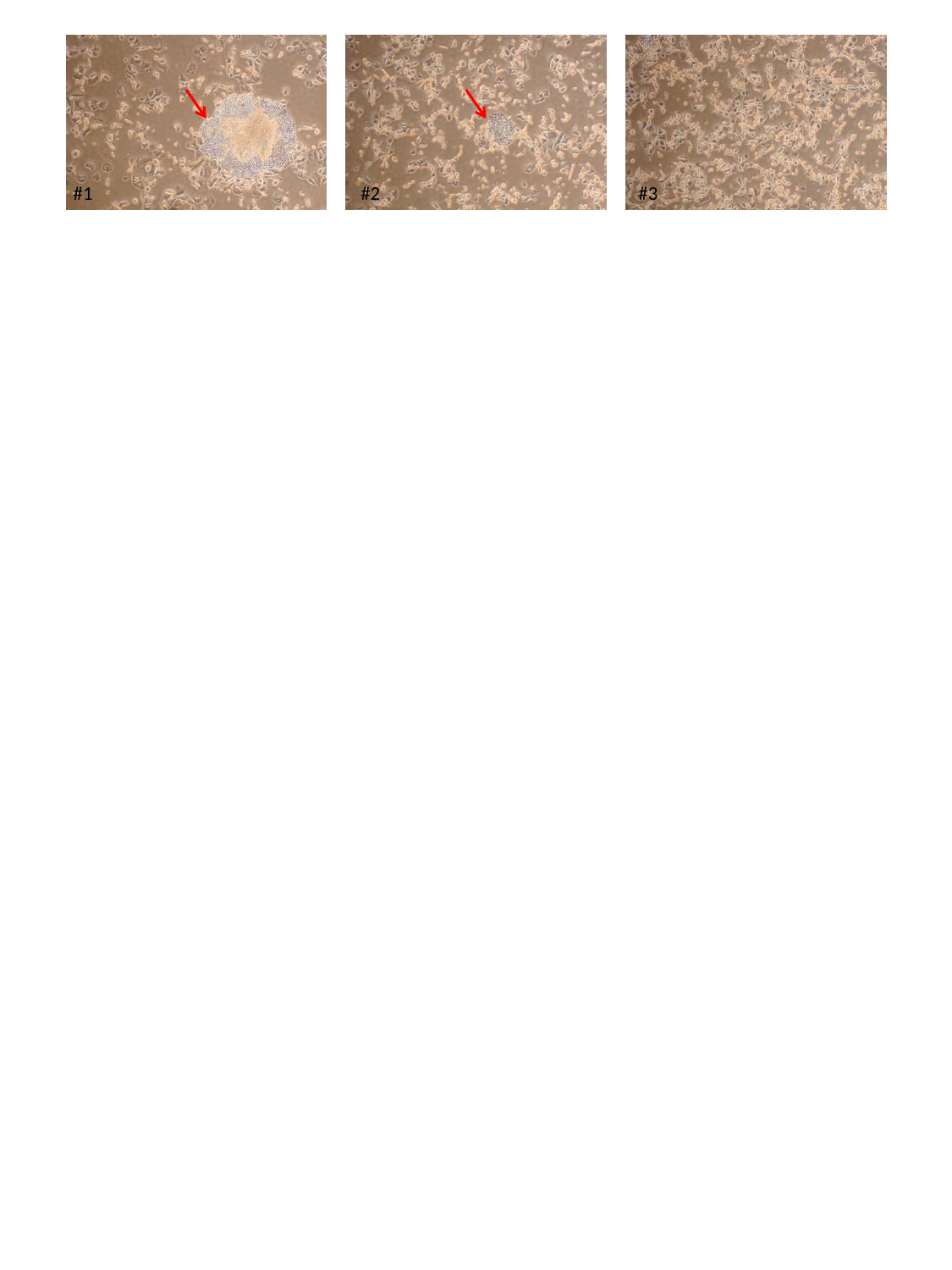

#1
#2
#3

Supplement: Additional file 1: Figure S1. — Optimization of the treatment schedule potentiates MTA-cisplatin anticancer efficacy. Representative image of a large H460 clone (indicated by the arrow after treatment #1) and a small clone (indicated by the arrow after treatment #2) at day 10 of the recovery phase. Quantification of clones was performed as described in the material and methods section. (PPTX 230 kb) [file 12885_2016_2117_MOESM1_ESM.pptx]

## Slide 1
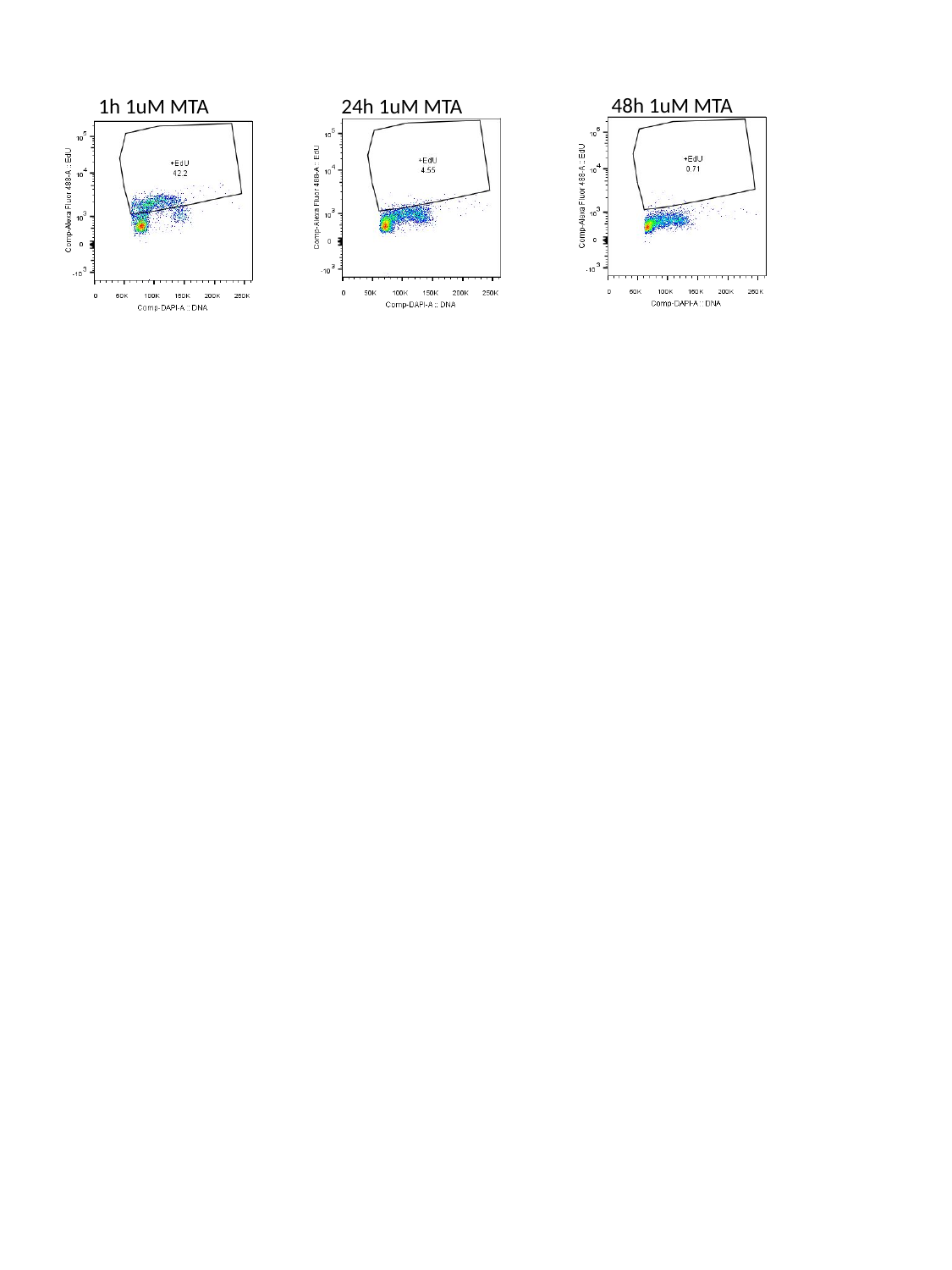

48h 1uM MTA
24h 1uM MTA
1h 1uM MTA

Supplement: Additional file 4: Figure S4. — MTA treatment abolishes DNA replication. Treatment of A549 cells with 1 μM MTA for the indicated time points. DNA replication, indicated by EdU incorporation, and cellular DNA content (DAPI) were detected simultaneously by flow cytometry. (PPTX 62 kb) [file 12885_2016_2117_MOESM4_ESM.pptx]

## Slide 1
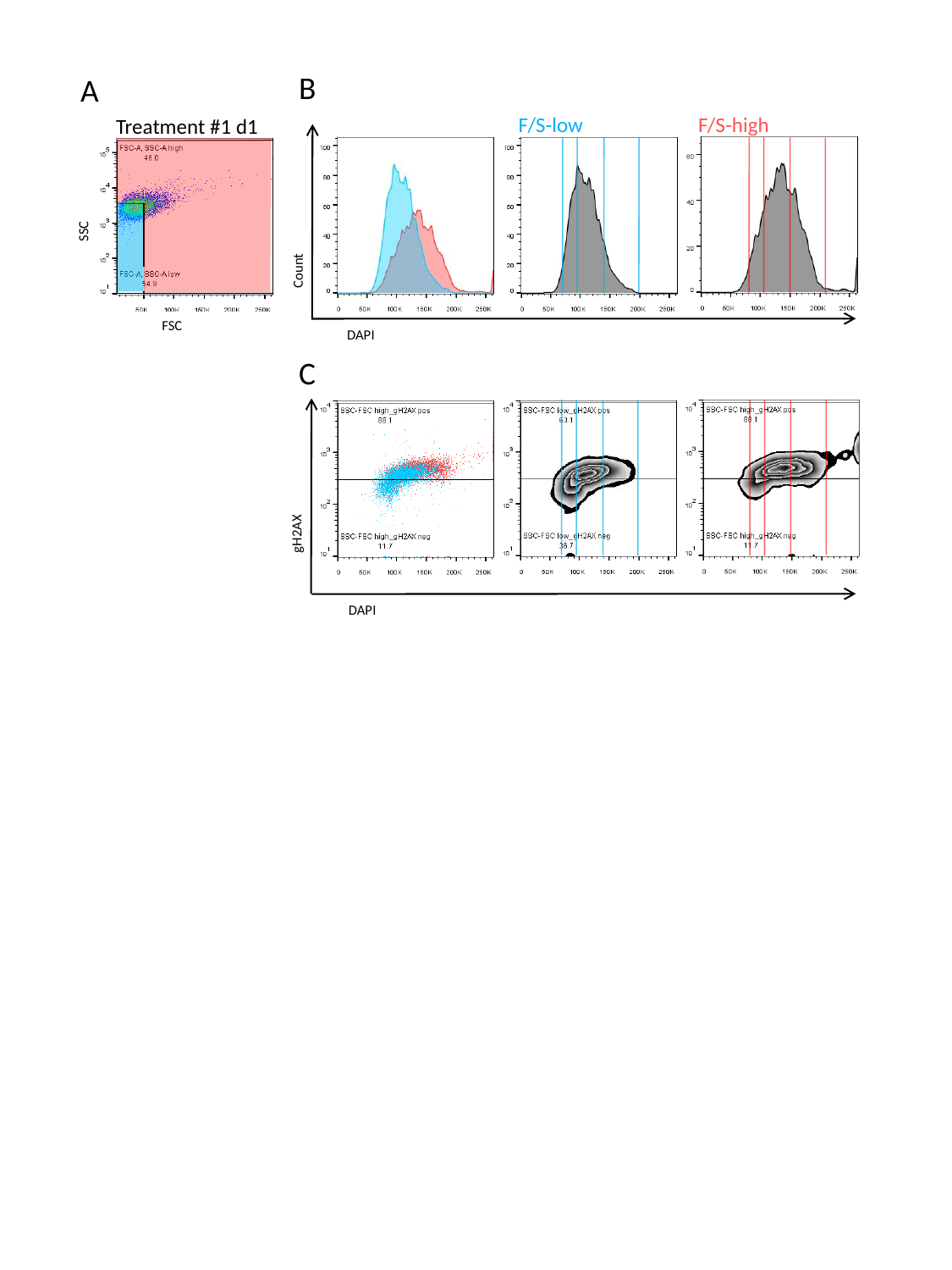

B
A
F/S-low
F/S-high
Treatment #1 d1
Count
DAPI
SSC
FSC
gH2AX
DAPI
C

Supplement: Additional file 5: Figure S5. — Applied strategy to determine cell cycle phases and cell cycle phase-specific H2AX phosphorylation levels by flow cytometry. To determine more accurately the G1 to S- and the S to G2/M-borders of the cell cycle phases from cell populations with irregular cell cycle distributions, H2AX phosphorylation over DNA content was blotted as “zebra blots” as described in the text. Shown are specific cell cycle gates indicated in blue for the F/S-low subpopulations of untreated controls. Specific cell cycle gates indicated in red are shown for the F/S-high subpopulations of treated samples. A 5 % threshold for basal H2AX phosphorylation levels was applied as indicated in the material and methods section. Analysis was performed after 24 h of MTA and cisplatin co-treatment, e.g. treatment #1, day 1. Data shown are representative of three experiments. (PPTX 104 kb) [file 12885_2016_2117_MOESM5_ESM.pptx]
