# Supplementary material for: Prolonged pemetrexed pretreatment augments persistence of cisplatin-induced DNA damage and eliminates resistant lung cancer stem-like cells associated with EMT
Source: BMC Cancer. 2016 Feb 19;16:125. doi: 10.1186/s12885-016-2117-4 (PMC4759918; doi:10.1186/s12885-016-2117-4)
Supplement: Additional file 2: Figure S2. — Flow cytometric analysis of forward (cell size) and side (cellular granularity) scatter intensity as an alternative readout for senescence. Approximately 5 % of the cells of the untreated controls were placed in the F/S-high compartment and used as normalization standard as described in the material and methods section. Forward and side scatter analysis by flow cytometry (without reseeding) at the indicated time points during the treatment and recovery phase. Shown are representative images of three experiments. (PPTX 389 kb) [file 12885_2016_2117_MOESM2_ESM.pptx]

## Slide 1
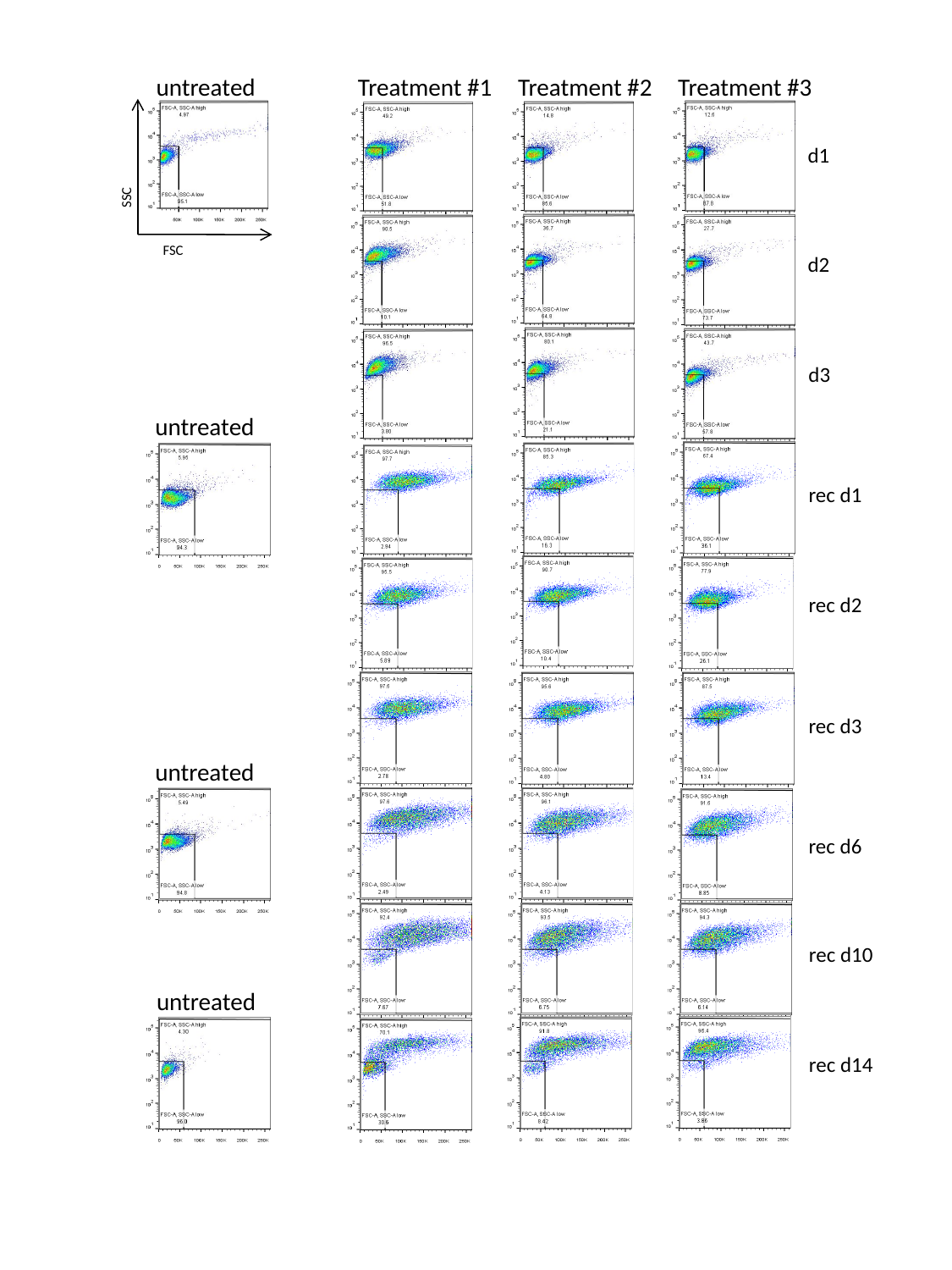

untreated
Treatment #1
Treatment #2
Treatment #3
d1
SSC
FSC
d2
d3
untreated
rec d1
rec d2
rec d3
untreated
rec d6
rec d10
untreated
rec d14
