# Supplementary material for: Prolonged pemetrexed pretreatment augments persistence of cisplatin-induced DNA damage and eliminates resistant lung cancer stem-like cells associated with EMT
Source: BMC Cancer. 2016 Feb 19;16:125. doi: 10.1186/s12885-016-2117-4 (PMC4759918; doi:10.1186/s12885-016-2117-4)
Supplement: Additional file 3: Figure S3. — MTA-cisplatin treatment leads to increased cell size (FSC) and granularity (SSC) thereby influencing DAPI signal intensity. A549 cells were concomitantly treated with MTA-cisplatin for 24 h and subsequently analyzed by flow cytometry. A Subpopulations featuring either normal forward and side scatter intensity (F/S-low) or increased forward and side scatter intensity (F/S-high) are indicated by the blue and red gates, respectively. B Histogram blots of DNA content from F/S-low and F/S-high subpopulations. Specific cell cycle phases of the F/S-low and F/S-high subpopulations are indicated by the blue and red gates, respectively. C Cell cycle phase-specific H2AX phosphorylation was determined by the same cell cycle gating strategy as shown in B in combination with applying the 5 % threshold for basal H2AX phosphorylation levels as indicated in the material and methods section. (PPTX 376 kb) [file 12885_2016_2117_MOESM3_ESM.pptx]

## Slide 1
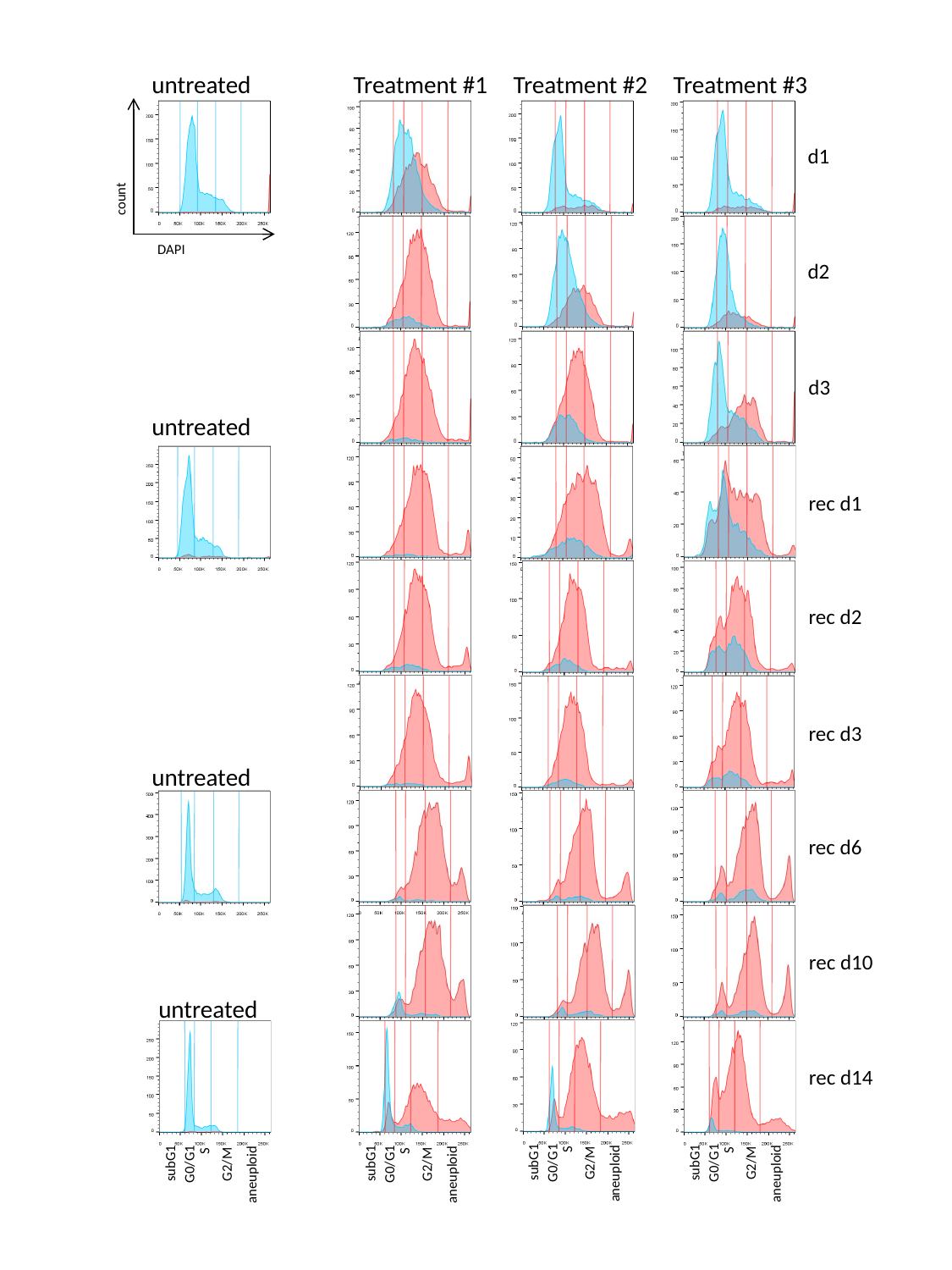

untreated
Treatment #1
Treatment #2
Treatment #3
d1
count
DAPI
d2
d3
untreated
rec d1
rec d2
rec d3
untreated
rec d6
rec d10
untreated
rec d14
subG1
G0/G1
S
G2/M
aneuploid
subG1
G0/G1
S
G2/M
aneuploid
subG1
G0/G1
S
G2/M
aneuploid
subG1
G0/G1
S
G2/M
aneuploid
