# Supplementary material for: Prolonged pemetrexed pretreatment augments persistence of cisplatin-induced DNA damage and eliminates resistant lung cancer stem-like cells associated with EMT
Source: BMC Cancer. 2016 Feb 19;16:125. doi: 10.1186/s12885-016-2117-4 (PMC4759918; doi:10.1186/s12885-016-2117-4)
Supplement: Additional file 6: Figure S6. — Applied strategy to determine cell cycle phases of subpopulations by flow cytometry. Flow cytometric analysis was performed at the indicated time points and subpopulations featuring either increased forward and side scatter intensity (F/S-high) or normal forward and side scatter intensity (F/S-low) were identified as indicated in Supplementary Figure S3. Cell cycle analysis was performed independently for F/S-low and F/S-high cells. Gates set to determine the cell cycle distribution of F/S-low and F/S-high subpopulations were determined as described in Figure S5 and are indicated in blue and red, respectively. Indicated are days (d) during treatment and the recovery phase (rec d). Data shown are representative of three experiments. (PPTX 386 kb) [file 12885_2016_2117_MOESM6_ESM.pptx]

## Slide 1
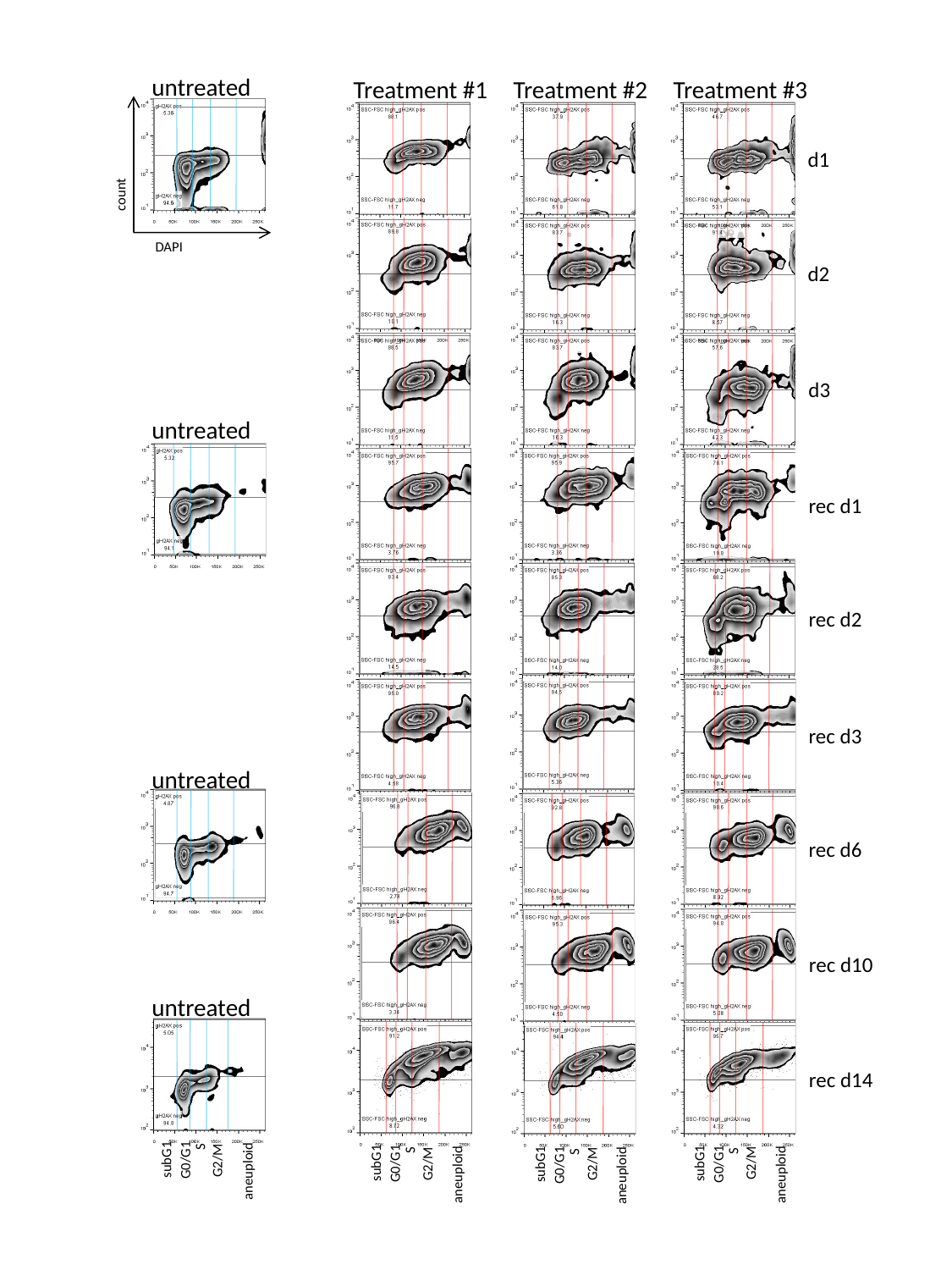

untreated
Treatment #1
Treatment #2
Treatment #3
d1
count
DAPI
d2
d3
untreated
rec d1
rec d2
rec d3
untreated
rec d6
rec d10
untreated
rec d14
subG1
G0/G1
S
G2/M
aneuploid
subG1
G0/G1
S
G2/M
aneuploid
subG1
G0/G1
G2/M
S
aneuploid
subG1
G0/G1
G2/M
S
aneuploid
